# Interactions between Personality and Types of Mindfulness Practice in Reducing Burnout in Mental Health Professionals

**DOI:** 10.3390/ijerph18136721

**Published:** 2021-06-22

**Authors:** Raquel Ruiz-Íñiguez, Ana Carralero Montero, Francisco A. Burgos-Julián, Justo Reinaldo Fabelo Roche, Miguel A. Santed

**Affiliations:** 1 Faculty of Psychology, National University of Distance Education, 28040 Madrid, Spain; rruiz555@alumno.uned.es (R.R.-Í.); psico.fburgos@gmail.com (F.A.B.-J.); 2 Department of Nursing, Faculty of Medicine and Health Sciences, University of Alcalá, 28805 Alcalá de Henares, Spain; a.carralero@uah.es; 3 Center for Academic Development in Drug Addiction (CEDRO), Universidad de Ciencias Médicas de la Habana, La Habana 10400, Cuba; fabelo@infomed.sld.cu

**Keywords:** meditation, mindfulness, personality, mental health, professionals, burnout

## Abstract

Research on mindfulness-based interventions reports mainly on improvements at the group level. Thus, there is a need to elaborate on the individual differences in their effectiveness. The aim of this study was twofold: (1) to examine which personality factors could influence burnout reduction associated with different types of mindfulness practice and (2) to evaluate the interaction between personality factors and the amount of home practice; both aims were controlled for sociodemographic characteristics. A total of 104 Cuban mental health professionals, who participated in a crossover trial, were included. The effect of personality (Cattell’s 16 Personality Factors) was analyzed through regression analysis. First, the results revealed that Emotional Stability and Vigilance could negatively moderate the effectiveness of mindfulness-based interventions. Second, participants who scored low in Sensitivity or Vigilance could benefit more from the body-centered practices (i.e., body scan and Hatha yoga practices), but no significant results for the mind-centered practices (i.e., classical meditation) were found. Third, participants who scored high in Self-reliance could benefit more from informal practice. Other personality factors did not appear to moderate the effect of the interventions, though previous experience in related techniques must be considered. Recommendations and clinical implications are discussed. Trial registration number is NCT03296254 (clinicaltrials.gov).

## 1. Introduction

Currently, around 46 million people in the US practice some type of meditation [1], being one of the most common methods of complementary and alternative medicine in the West. Mindfulness meditation is one of the modalities that is gaining in importance. Mindfulness is a human capacity that has been described as “paying attention in a particular way: on purpose, in the present moment, and nonjudgmentally” (Kabat-Zinn, 1994, p.4) [2]. Mindfulness-based interventions (MBIs) comprise a diversity of meditation practices that aim to expand this capacity. Most are based on Mindfulness-Based Stress Reduction (MBSR), the first program to standardize mindfulness techniques. Several components and practices are included in MBIs, a multidimensionality that presents difficulty for mindfulness research [3]. Dismantling studies are a key to the deep exploration of the different components of MBIs [4]. MBSR includes three formal practices (i.e., meditation, body scan and Hatha yoga) and informal practices (which consist of bring mindful awareness to everyday activities, such as eating or washing dishes). Mindfulness meditation integrates two kinds of mental practices: focused attention meditation (in which the practitioner is required to focus attention on a chosen object or event, such as breathing or a sound) and open monitoring meditation (in which the focus of the meditation becomes the monitoring of awareness itself; there is no object or event to focus on). Body scan is the first exercise taught in MBIs and consists of paying attention to parts of one’s body and bodily sensations in a gradual sequence. Hatha yoga in MBIs consists of mindful movements and stretching. Body scans and Hatha yoga are strongly related, since both are a way of observing bodily sensations where there is complete contact of the body with the physical world [5]. In this study, mindfulness meditations will be denominated as mind-centered practices in order to distinguish from body scan and Hatha yoga, which will be denominated as body-centered practices. A standard MBI lasts eight weeks and requires 40–45 min of daily home practice; a brief MBI has a duration of 30 min or less in home practice and lasts up to 4 weeks [6]. In health professionals, a lack of time makes brief interventions ideal [7]. Studies on MBIs for healthcare professionals have indicated their effectiveness in reducing burnout, stress, depression and anxiety, among others [8,9,10]. However, for advancing towards personalized assistance, it is essential to deeply examine some aspects of the effectiveness of MBIs. Individual differences may likely contribute to the way people respond to and benefit from mindfulness practices [11], and different types of mindfulness practices could benefit certain types of people more than others [12]. Previous experience in meditation has been identified as another relevant issue to consider [13]. Research on the association between mindfulness and personality traits has mainly studied mindfulness traits, also called dispositional mindfulness [14].

Individual variability in the effectiveness of mindfulness training has not been systematically investigated [15]. However, personality traits play a key role in influencing the effects of psychological interventions. Studies have pointed out that previous high levels of neuroticism lead to better results for aspects such as anxiety, depression, mental distress or subjective well-being [16,17]. Conscientiousness did not show significant moderating effects in one study [17], but another study indicated that higher scores on conscientiousness showed an increased effect of mindfulness training on stress reduction [16]. Extroversion and openness to experience did not moderate the effects of the intervention; agreeableness was not assessed [16,17].

Although mindfulness instruction includes a strong commitment to home practice, a review in which controlled studies of mindfulness-group interventions were analyzed showed mixed results regarding the relation between home practice and clinical outcomes [18]. Moreover, a recent meta-analysis of MBSR and mindfulness-based cognitive therapy (MBCT) showed a significant but small association between the extent of practice and positive outcomes [19]. To date, little monitoring of this topic has been done in research, and there are few studies that have analyzed the association between the amount of practice and personality traits, but without relating them to the effectiveness of the interventions [20,21]. As shown above, and in order to advance in the development of MBIs, it is necessary to go beyond establishing their effectiveness. Thus, the first aim of this study was to examine which personality factors could influence burnout reduction associated with two brief MBIs, controlling for socio-demographic factors (age, sex and previous experience in related techniques), in Cuban mental health professionals. The second aim was to evaluate the interaction between personality factors and the amount of home practice (both formal and informal practice) in the effectiveness of both interventions, controlling for the same socio-demographic factors mentioned above. Given the literature review described above, the moderating effects of personality on burnout reduction of both MBIs were hypothesized for pre-intervention levels of personality factors. Neuroticism was hypothesized to be positively associated with burnout reduction. Considering the arguments above, the expectations for the other personality dimensions were null, rendering the tests on these traits mostly explorative in nature. To our knowledge, the moderating role of the personality, depending on the type of practices (mind/body-centered) or on the amount of practice (formal/informal), in the effectiveness of MBIs have not been explored until now, so this was also an exploratory hypothesis. Finally, regarding the control variables, previous experience was hypothesized to modulate the burnout reduction; for people with previous experience in meditation and related techniques, both interventions will be more effective compared to people without experience. For gender and age, it was hypothesized that they would have no influence on the effectiveness of both MBIs in burnout reduction.

## 2. Materials and Methods

This study was based on the analysis of part of the data collected in a randomized controlled trial that investigated the differential efficacy of two brief MBIs for reducing anxiety, work stress and burnout. Participants were randomized to one of the two experimental groups. Each experimental group received the two interventions, one after the other, but in reverse order, in a crossover design. This study tried to explore one particular area, i.e., personality traits.

This study evaluated those dependent variables that had demonstrated their effectiveness for both groups at the crossover point, resulting in burnout syndrome as the variable to be analyzed (there were no significant results on anxiety and work stress at the crossover point for both groups).

This study was approved by the Ethics Committee of the General Calixto García Faculty of the University of Medical Sciences of Havana (2018, page 168). It was conducted in accordance with the ethical standards of the Helsinki Declaration of 1975 and its later amendments. Informed consent was obtained from all individual participants included in the study.

### 2.1. Participants

Mental health professionals in Havana (Cuba) were contacted to participate in free mindfulness training through a call by Infomed (digital portal of the health network in Cuba) and by mailing lists to the main referents in the field of mental health in the area. The two interventions took place from January to March 2018. The inclusion criteria were (a) being a mental health professional, (b) currently working in an assistance practice, and (c) having a commitment to comply with the intervention. People who had some type of medical or mental condition that did not recommend their participation were excluded.

Of the 130 professionals who enrolled, 5 did not meet the eligibility criteria and 21 did not attend, resulting in a final sample size of 104 subjects (93 women, 10 men, one unspecified), aged 20–66 (*M* = 41 years, *SD* = 11.91). Regarding previous experience, participants were novices in mindfulness meditation, but 36.9% had experience in related techniques (i.e., transcendental meditation, Tai chi, Chi-kung and yoga). Hypnosis and Reiki were ruled out as previous experience. According to the analysis of statistical power, this sample size was sufficient to achieve a high effect size (*f*^2^ = 0.35), with a power of 0.80 and an alpha level of 0.05.

All study participants were kept anonymous, and they signed an informed consent to participate in the research. The participants did not receive any compensation for their participation.

### 2.2. Interventions

The instructor of the interventions (first author), with a great deal of experience in mindfulness (>seven years), was certified by the Spanish Association of Mindfulness and Compassion. A professional with recognized qualifications (last author) and a great deal of experience as a trainer and instructor (>13 years) supervised the intervention.

One brief mindfulness intervention was based on body-centered practices (i.e., Hatha yoga and body scan). The other brief mindfulness intervention was based on mind-centered practices (i.e., focused attention and open monitoring meditation).

Each intervention was based on a five-week training schedule, with one session/week of 2.5 h, and with recommended home practices of 20 min/day for the rest of the week. Thus, each participant received a total of 10 weeks of mindfulness training. There was no retreat day. Audio guides were provided by the instructor for formal home practice.

### 2.3. Measurements

#### 2.3.1. Sociodemographic Data

Participants responded to items regarding their age, sex, and previous experience in related practice. This open question was asked to find out if the subjects had previous experience in mindfulness, other types of meditation practice or related practices: *What techniques or similar practices have you carried out?* The answers were subsequently dichotomized into *Yes/No.*

#### 2.3.2. Burnout

The Brief Burnout Questionnaire (BBQ-R; [22]) is an adaptation of the Maslach Burnout Inventory, and a content validation process in Cuba [23] was used. It is made up of 21 items that evaluate burnout syndrome, in addition to other aspects (i.e., antecedents and consequences of burnout). However, this scale does not allow a differentiated measurement of its three dimensions (emotional exhaustion, depersonalization, and personal accomplishment). The answers are based on a Likert scale (from 1: *Never or Nothing* to 4: *Always or A lot*). The burnout syndrome is categorized as low (9–19 points), medium (>19–25 points) and high (>25 points). For the analysis of this study, the burnout syndrome offered a McDonald’s omega of 0.82, a coefficient recommended when the data is ordinal [24].

#### 2.3.3. Personality Factors

Catell and Eber’s 16-personality factor inventory (16-PF; [25]) was used in its Form C (which includes an emotional distortion scale). It was adapted to the Cuban population [26]. It consists of 105 items, with three alternatives in the answers, which vary according to the question (true/yes/option1, doubtful/sometimes and false/no/option 2). The answers can be scored as 2, 1 or 0 points. From the gross scores, weighted scores are obtained from 1 to 10, defining two opposite poles for each factor (low, 1–3 scores; high, 8–10 scores). Although the questionnaire measures 16 personality factors, only those factors with a McDonald’s omega greater than 0.65 were considered for analysis, which is considered as minimally acceptable. These factors were A (warmth, 0.76), C (emotional stability, 0.67), E (dominance, 0.68), F (liveliness, 0.69), I (sensitivity, 0.71), L (vigilance, 0.71), M (abstractedness, 0.74), Q2 (self-reliance, 0.69), Q3 (perfectionism, 0.69) and Q4 (tension, 0.75). The other factors not considered in this study were G (rule-consciousness), H (social boldness), N (privateness), O (apprehension) and Q1 (openness to change). For the same reason, the second-order factors of the model were not considered (factor QI adaptation-anxiety, factor QII introversion-extraversion, factor III emotionality-dynamism and factor IV submission-independence and neuroticism).

#### 2.3.4. Home Practice

A practice log delivered weekly was used, where participants had to fill in each exercise practiced and the time devoted to it each day (in the case of informal practices, they were asked for an estimate of the time devoted to them). The amount of practice performed in total minutes during the five weeks of training was quantified. A differentiation was made between formal and informal practice.

A conservative criterion was used for the analysis of the practice log to avoid the overestimation of data that the imputation would entail. Thus, non-delivery of a practice log for a week was counted as zero practices that week, following the recommendations in this regard [27]. The reliability and validity of this type of records has been established for home practices in MBSR [20].

### 2.4. Data Analysis

First, a hierarchical multiple regression analysis was performed. Post hoc effects were studied for each of the interactions and were significant via computation of the simple slopes. Second, a moderated hierarchical multiple regression analysis was carried out for each of the two intervention groups. Reliability analysis using the test-retest procedure was based on the intraclass correlation coefficient (ICC). Values less than 0.5 indicated poor reliability, values between 0.5 and 0.75 indicated moderate reliability, and values between 0.75 and 0.9 indicated excellent reliability [28].

The values of personality factors were centered on the grand mean to reduce the effects of multicollinearity. Variance inflation factor (VIF) values were calculated for each step, where values ≥ 10 would indicate multicollinearity problems. The coefficient of determination (R^2^) was calculated to check the fit of the models.

Statistically significant results were assumed for a two-sided *p*-value < 0.05 or absence of the zero value in the 95% confidence interval. Statistics were done using R 3.5.0. The emmeans (v 1.15-15) and the apaTablet (v 2.0.5) packages were used. The full computer code is available in the Supplementary Online Materials.

## 3. Results

Means, standard deviations and Pearson correlations for quantitative variables are shown in the  Supplementary Online Materials (see Table S1). There was 10.6% of subjects with a high level of burnout, 38.4% with a medium level, and 51% with a low level. Burnout reduction was weakly associated with *Emotional stability* (*r* = −0.22, *p* = 0.025), *Dominance* (*r* = 0.24, *p* = 0.012) and *Vigilance* (*r* = −0.25, *p* = 0.011).

### 3.1. Main Regression Analyses

A hierarchical multiple regression analysis was carried out to respond to the first objective of the study. To do this, as a first step, the block of sociodemographic variables was introduced as control variables. As a second step, the main effects of personality factors were introduced into the model. As a third step, a moderated regression model was built, introducing the interaction terms between group and personality factors.

Table 1 shows the results of the hierarchical multiple regression analysis. The data referring to the variables that turned out to be non-significant predictors in model 3 were omitted (see Supplementary Online Materials, Table S2, for these details). The VIF values ranged between 1.12 and 2.46, which indicated the absence of multicollinearity problems.

Model 1 achieved a significant fit (*R*^2^= 0.11, *p* = 0.04). When progressing to model 2, the coefficient of determination was also significant (*R*^2^ = 0.39, *p* < 0.01). In this model, the personality traits *Emotional Stability* (*b* = −0.52, *SE* = 0.25, *p* = 0.04, 95% CI (−1.02, −0.02)) and *Vigilance* (*b* = −0.59, *SE* = 0.20, *p* = 0.02, 95% CI (−2.91, −0.01)) significantly predicted burnout reduction. When progressing to model 3, the coefficient of determination was significant (*R*^2^ = 0.59, *p* < 0.01), with a significant interaction between the intervention group and the personality traits *Sensitivity* (*b* = 1.51, *SE* = 0.48, *p* = 0.01, 95% CI (0.54, 2.48)), *Vigilance* (*b* = 1.28, *SE* = 0.41, *p* = 0.01, 95% CI (0.45, 2.11)), and *Tension* (*b* = −0.99, *SE* = 0.45, *p* = 0.03, 95% CI (−1.90, −0.08)).

In those interactions that were significant in model 3, the effects of simple slopes were analyzed post hoc. Thus, a significant effect was found for the body-centered practices group and the personality traits *Sensitivity* (*b* = −1.19, *SE* = 0.32, 95% CI (−1.83, −0.55)) and *Vigilance* (*b* = −1.35, *SE* = 0.32, 95% CI (−1.99, −0.71)). In the interaction between this group and *Tension,* the effect of the simple slope did not show a significant relationship (*b* = −0.10, *SE* = 0.30, 95% CI (−0.71, 0.50)). In the mind-centered practices group, no significant effects were obtained for any of the personality traits analyzed: *Sensitivity* (*b* = 0.49, *SE* = 0.26, 95% CI (−0.04, 1.02)), *Vigilance* (*b* = −0.02, *SE* = 0.27, 95% CI (−0.56, 0.52)) or *Tension* (*b* = −0.11, *SE* = 0.36, 95% CI (−0.82, 0.59)).

### 3.2. Secondary Regression Analyses

A moderated hierarchical multiple regression analysis for each of the two groups was carried out to respond to the second objective of the study. Like in the main analysis, as a first step, the block of sociodemographic variables was introduced as control variables. As a second step, the main effects of the personality variables were introduced into the model and, for those that were significant, their interaction with the amount of home practice (both formal and informal) was measured. The VIF values ranged between 1.06 and 2.68, indicating the absence of multicollinearity problems. Furthermore, the results showed moderate reliability (ICC = 0.74; *p* < 0.01; 95% CI (0.62, 0.87)). Table 2 and Table 3 show the results of the multiple regression analyses for each intervention group.

For the body-centered practices group, in step 1, a significant fit was not reached (*R*^2^ = 0.05, *p* = 0.45). In step 2, the goodness of fit was significant (*R*^2^ = 0.63, *p* < 0.01). This was also the case in step 3 (*R*^2^ = 0.25, *p* = 0.24), in which there was a significant interaction with the minutes of informal practice for the personality traits *Vigilance* (*b* < −0.01, *SE* < 0.01, *t* = −2.17, *p* = 0.04, 95% CI (−0.01, 0.00)) and *Self-reliance* (*b* = 0.004, 0.01, *SE* < 0.01, *t* = 3.05, *p* < 0.01, 95% CI (0.001, 0.006)). In the moderation analysis for formal practices, no significant moderating effects were obtained (see Supplementary Online Materials, Table S3, for further details).

For the mind-centered practices group, the hierarchical regression model in step 1 showed a significant fit (*R*^2^ = 0.16, *p* = 0.03), with a main effect on the variable previous experience in related techniques (*b* = 3.55, *SE* = 1.24, *t* = 2.86, *p* < 0.01, 95% CI (1.06, 6.04)). In step 2, no significant fit was obtained (R^2^ = 0.33, *p* = 0.17), and there were no significant main terms, so step 3 was not continued.

## 4. Discussion

This study examined personality factors as putative moderators of the effectiveness of two brief mindfulness interventions (one focusing on body-centered practices, the other focusing on mind-centered practices) in reducing burnout levels in mental health professionals, controlling for three socio-demographic factors (age, sex and previous experience in related techniques).

Regarding the first aim of the study, our results in the main regression analyses (Model 2) revealed that the personality factors *Vigilance* and *Emotional stability* were negatively associated with burnout reduction in brief MBIs. This means that individuals in the low range of the *Vigilance* scale who are more inclined to be trusting (unsuspecting, accepting, unconditional, easy) and individuals in the low range of the *Emotional stability* scale, who are more inclined to be high-strung (reactive emotionally, changeable, affected by feelings, easily upset) benefited more. Our results in the main regression analyses (Model 3: interaction) revealed that *Sensitivity* positively moderated the effectiveness of body-centered practices (i.e., Hatha yoga and body scan). This means that individuals in the high range of the *Sensitivity* scale, who have the greatest tendency to be tough-minded (utilitarian, self-reliant, no-nonsense, objective) benefited more from body-centered practices. No moderating results were found for specific personality factors for mind-centered practices (i.e., classical sitting meditation).

In order to be able to compare these results, it is necessary to know the equivalence with the Five Factor Model (FFM). Rossier et al. [29] analyzed the similarity between Cattell’s 16-PF scale and the FFM measured by NEO Personality Inventory Revised (NEO PI-R). They found that *Neuroticism* (NEO PI-R) correlated negatively with *Emotional stability* (16-PF), and *Agreeableness* (NEO PI-R) correlated negatively with *Vigilance* (16-PF). None of the NEO PI-R scales correlated well with *Sensitivity* and *Self-reliance* (correlations below 0.40).

Our study is consistent with prior findings, in which *Neuroticism* moderated effects on stress reduction and the related constructs [16,17]. Therefore, MBIs would work better in a neurotic personality profile, as the capacity for emotional regulation is improved [16]. Individuals with high initial levels of *Neuroticism* have been reported to have a more negative mood [17], and brief mindfulness interventions would be more effective for them since they perceive the greatest improvement in emotion processing [30]. On the other hand, *Agreeableness* was shown to be significant in our study, in contrast to the assumption that this trait seemed less relevant [17]. However, it has been noted that people with high *Agreeableness* scores are more likely to be more compliant in the short term [20], which would be consistent with our results. They also value cooperation and social harmony, feeling comfortable and accepted by social groups [31], which would be in line with our group setting training. Finally, being trusting (low *Vigilance*) or tough-minded (low *Sensitivity*) at baseline would make a person participate more easily, be more open to the content and exercises implemented, and have a greater commitment from the start, which would be more important in body-centered practices. What is more, the absence of moderating personality results in the mind-centered practices indicated that the impact of certain personality traits would be different for body-centered vs. mind-centered practices, with greater influence of the first. We have not found evidence in the literature that can explain this difference.

Regarding the second aim of the study, our results in the secondary regression analyses examining body-centered practices (Step 3) indicated that *Vigilance* (negatively) and *Self-reliance* (positively) moderated the effectiveness of informal practice in reducing burnout. This means that individuals in the low range of the *Vigilance* scale, who have the greatest tendency to be trusting, and individuals in the high range of the *Self-reliance* scale, who are more inclined to be self-sufficient (self-reliant, solitary, resourceful, individualistic), benefited more from the amount of informal practice done during an MBI of body-centered practices.

The importance of being trusting and self-reliant when practicing informal mindfulness makes sense when related to the characteristics of the informal practice: they are free (unguided), autonomous and individual practices. Maybe trusting and self-reliant individuals feel more comfortable doing informal practices, thus feeling more effective. Each person integrates them into their personal life in their own way, by choosing what exercise to do, when to do it and for how long [32] and trusting in their own ability to carry it out without being guided. These results are in line with a study on MBSR that concluded that *Agreeableness* predicted greater use of meditation practice during the intervention [20]. However, this research did not differentiate between formal and informal practice, unlike our study.

Regarding the role of control variables (age, sex and previous experience) presented in the two objectives, our results in the main regression (Model 1) indicated that previous experience in related techniques was associated with burnout reduction. So, more experienced individuals benefited more from MBIs, which is consistent with previous studies [33,34]. DeVibe et al. [16] suggested that this could be related to a higher level of previous trait/dispositional mindfulness in experienced people. The secondary regression analysis examining mind-centered practices (Step 1) indicated that it would be particularly beneficial in mind-centered practices (i.e., classical meditation). We did not find evidence in the literature that could explain why it is maintenance only in mind-centered practices but not in body-centered practices. Moreover, it should be noted that these effects disappeared when personality was introduced into the models. The absence of significance for sex is consistent with previous studies [17,35], but contrasts with de Vibe et al. [16], who noted that women benefit significantly more from MBIs, as they have higher neuroticism levels than men. Finally, age did not predict changes in effectiveness in our study, where previous studies presented contradictory results [17,35].

### Limitations and Future Directions

Caution is recommended in interpreting our findings due to the limitations of this study. The lack of statistically significant association with other personality traits could be due to relatively low initial levels of burnout (floor effect), with 51% of the sample presenting a low level, or to the lack of inter-individual variation in the measures of personality factors. On the other hand, the results of our study could also be limited by a sample size that was too modest to reach statistical significance in the secondary regression analysis, when dividing the sample into two groups. The sample has very specific characteristics: Cuban mental health professionals from Havana, being mainly women in adulthood, which may limit the degree to which our results can be generalized. A more diverse sample could give different results. In addition, there may be a bias in the sampling of the participants, due to a self-selection of people open to the idea of participating in this type of intervention. In future research, efforts should be made to diversify the participants in terms of gender, age, occupation, regional demographics and previous interest, and to have a control group. Considering these contradictions and the lack of evidence to provide a consistent explanation about the role of the control variables, further extensive research is necessary in order to clarify the interaction between personality factors and formal/informal mindfulness practice.

Currently, the Big Five Model is the most used in personality research. This model could not be used in this research, as the widely used questionnaires used in its development (such as the NEO-PI or NEO-FFI) have not been validated for the Cuban population; thus, the comparison with previous personality studies was limited. For the same reason, dispositional mindfulness levels could not be measured. Future research should incorporate this measurement and delve more deeply into the differential effect of personality, depending on the types of mindfulness practice included. In response to our results, future research on MBIs should consider the possible relevant role of *Agreeableness,* usually not taken into account in this type of study. A more in-depth study is necessary to develop hypotheses about why personality traits impact differentially between different mindfulness practices.

Regarding the analysis of home practice, there are also some limitations to consider. The quality of the practice was not assessed, and the measurement of the quantity may have inaccurate estimates, especially in informal practice, due to the difficulties inherent in self-reporting measures. There is also a risk that the data presented may be overestimated because of desirability or social adequacy regarding compliance with what was recommended by the facilitator. In our study, we attempted to compensate for this by underestimating the missing data, using a conservative accounting criterion. Future research should measure using a more objective method than self-reporting, such as smart phone technology. Participants should not only measure the quantity but also the quality of the practice, and the relationship between the two, in order to draw more precise conclusions. Further research regarding the role of informal vs. formal mindfulness practice and personality traits in the effectiveness of MBIs is encouraged.

If individual differences do indeed significantly contribute to mindfulness training outcomes, then such information would be crucial to enhance their overall effectiveness and cost-effectiveness. Measurements of personality traits before selecting a specific intervention could help guide therapists into a more effective intervention, correlating the type of practice to the personality of the individual seeking help. Emotionally stable people may improve more from other interventions. Some people would need additional training or treatment, particularly those who are suspicious (high vigilance), tender-hearted (high sensitivity), or dependent (low self-reliance). Taking these considerations into account would also allow existing mindfulness programs to be tailored to individual characteristics. In light of these lines of evidence, we advocate for an individual difference perspective in mindfulness training clinical practice.

## 5. Conclusions

Each person is unique, and MBIs should be tailored to specific contexts and individuals. Along this line, this study represents an effort to clarify which personality profiles in mental health professionals would benefit the most from two brief interventions for burnout reduction based on mind/body-centered practices. Our findings lead to a preliminary conclusion regarding the effectiveness of MBIs, with a negative moderating influence of *Emotional Stability*, *Vigilance* and *Sensitivity* in MBIs (the latter, only in body-centered practices), and the positive influence of *Self-reliance* on the impact of informal practice. The theoretical grounds for the moderating effect of the traits highlighted in this study need to be explored further.

## Figures and Tables

**Table 1:** Hierarchical multiple regression analysis and moderated regression (interaction: group) examining burnout reduction (method: stepwise).

Predictor	*b*	*b* 95% CI (LL, UL)	*beta*	*beta* 95% CI (LL, UL)	*sr* ^2^	*sr*^2^ 95% CI (LL, UL)	*r*	Fit
**Model 1**								
(Intercept)	0.67	(−5.40, 6.75)						
Age	0.03	(−0.05, 0.11)	0.08	(−0.14, 0.31)	0.01	(−0.03, 0.04)	0.10	
Sex	−1.31	(−4.12, 1.50)	−0.11	(−0.33, 0.12)	0.01	(−0.03, 0.06)	−0.12	
Previous experience ^a^	0.91 *	(0.19, 1.62)	0.29	(0.06, 0.52)	0.08	(−0.04, 0.20)	0.30 **	
								*R*^2^ = 0.109 *
**Model 2**								
(Intercept)	3.42	(−2.71, 9.56)						
Warmth	0.15	(−0.35, 0.65)	0.07	(−0.17, 0.32)	0.00	(−0.02, 0.03)	0.00	
Emotional stability	−0.52 *	(−1.02, −0.02)	−0.24	(−0.47, −0.01)	0.05	(−0.03, 0.12)	−0.15	
Dominance	0.34	(−0.10, 0.78)	0.18	(−0.05, 0.41)	0.02	(−0.03, 0.08)	0.21	
Liveliness	−0.27	(−0.71, 0.17)	−0.14	(−0.36, 0.08)	0.02	(−0.03, 0.06)	−0.14	
Sensitivity	−0.27	(−0.73, 0.20)	−0.14	(−0.38, 0.10)	0.01	(−0.03, 0.06)	−0.10	
Vigilance	−0.59 **	(−1.00, −0.19)	−0.31	(−0.52, −0.10)	0.09	(−0.02, 0.19)	−0.30 *	
Abstractedness	0.56	(−0.00, 1.11)	0.24	(−0.00, 0.48)	0.04	(−0.03, 0.11)	0.20	
Self-reliance	0.28	(−0.27, 0.84)	0.11	(−0.11, 0.34)	0.01	(−0.03, 0.05)	0.11	
Perfectionism	−0.21	(−0.74, 0.31)	−0.09	(−0.33, 0.14)	0.01	(−0.02, 0.04)	−0.14	
Tension	−0.34	(−0.81, 0.14)	−0.18	(−0.42, 0.07)	0.02	(−0.03, 0.07)	−0.05	
								*R*^2^ = 0.388 **
**Model 3** ^**b**^								
(Intercept)	8.59 *	(0.99, 16.19)						
Group × Sensitivity	1.51 **	(0.54, 2.48)			0.08	(−0.00, 0.17)		
Group × Vigilance	1.28 **	(0.45, 2.11)			0.08	(−0.00, 0.16)		
Group × Tension	−0.99 *	(−1.90, −0.08)			0.04	(−0.02, 0.10)		
								*R*^2^ = 0.593 **

Notes: *b* represents unstandardized regression weights; *beta* indicates the standardized regression weights; *sr*^2^ represents the semi-partial correlation squared; *r* represents the zero-order correlation; LL and UL indicate the lower and upper limits of a confidence interval, respectively. A significant *b*-weight indicates the beta-weight and semi-partial correlation are also significant. * indicates *p* < 0.05. ** indicates *p* < 0.01. ^a^ Previous experience in related techniques was represented as a dummy variable, with 1 serving as meditation-naïve subject. ^b^ Only the significant interactions of the model are indicated.

**Table 2:** Moderated hierarchical multiple regression analysis (interaction: amount of home practice) examining burnout reduction for body-centered practices group (method: stepwise).

Predictor	*b*	*b* 95% CI (LL, UL)	*beta*	*beta* 95% CI (LL, UL)	*sr* ^2^	*sr*^2^ 95% CI (LL, UL)	*r*	Fit
**Step 1**								
(Intercept)	3.07	(−4.60, 10.74)						
Age	−0.04	(−0.14, 0.06)	−0.12	(−0.40, 0.17)	0.01	(−0.08, 0.05)	−0.11	
Sex	−0.93	(−3.96, 2.10)	−0.09	(−0.38, 0.20)	0.01	(−0.05, 0.04)	−0.11	
Previous experience ^a^	1.37	(−0.94, 3.68)	0.17	(−0.12, 0.46)	0.03	(−0.12, 0.06)	0.18	
								*R*^2^ = 0.054
**Step 2**								
(Intercept)	16.81 **	(7.49, 26.12)						
Warmth	0.16	(−0.34, 0.65)	0.08	(−0.18, 0.35)	0.00	(−0.03, 0.02)	0.16	
Emotional stability	−0.64 *	(−1.18, −0.10)	−0.28	(−0.52, −0.05)	0.06	(−0.14, 0.02)	−0.19	
Dominance	0.40	(−0.10, 0.89)	0.20	(−0.05, 0.45)	0.03	(−0.08, 0.03)	0.27	
Liveliness	−0.41	(−0.93, 0.10)	−0.19	(−0.42, 0.05)	0.03	(−0.08, 0.03)	−0.02	
Sensitivity	−0.33	(−0.78, 0.13)	−0.16	(−0.39, 0.06)	0.02	(−0.07, 0.03)	−0.25	
Vigilance	−1.24 **	(−1.70, −0.79)	−0.65	(−0.88, −0.41)	0.31	(−0.49, −0.12)	−0.53 **	
Abstractedness	−0.07	(−0.57, 0.44)	−0.03	(−0.25, 0.19)	0.00	(0.01, 0.01)	0.04	
Self-reliance	0.87 *	(0.19, 1.55)	0.35	(0.08, 0.62)	0.07	(−0.16, 0.02)	0.26	
Perfectionism	−0.13	(− 0.71, 0.45)	−0.05	(−0.30, 0.19)	0.00	(−0.02, 0.01)	−0.23	
Tension	0.01	(−0.44, 0.46)	0.01	(−0.24, 0.26)	0.00	(−0.00, 0.00)	<0.001	
								*R*^2^ = 0.627 **
**Step 3** ^**b**^								
(Intercept)	1.69	(−0.34, 3.72)						
Informal practice × Vigilance	−0.002 *	(−0.004, 0.000)			0.11	(−0.29, 07)		
Informal practice × Self-reliance	0.004 **	(0.001, 0.006)			0.22	(−0.45, 0.02)		
								*R*^2^ = 0.253 *

Note: A significant *b*-weight indicates the beta-weight and semi-partial correlation are also significant. *b* represents unstandardized regression weights. *beta* indicates the standardized regression weights. *sr*^2^ represents the semi-partial correlation squared. *r* represents the zero-order correlation. LL and UL indicate the lower and upper limits of a confidence interval, respectively. In the third step, only those significant interaction terms are reported. The intercept is omitted from the model. * indicates *p* < 0.05. ** indicates *p* < 0.01. ^a^ Previous experience was represented as two dummy variables, with 1 serving as meditation-naïve subject. ^b^ Only the significant interactions of the model are indicated.

**Table 3:** Moderated hierarchical multiple regression analysis (interaction: amount of home practice) examining burnout reduction for mind-centered practices group (method: stepwise).

Predictor	*b*	*b* 95% CI (LL, UL)	*beta*	*beta* 95% CI (LL, UL)	*sr* ^2^	*sr*^2^ 95% CI (LL, UL)	*r*	Fit
**Step 1**								
(Intercept)	−8.52	(−22.14, 5.11)						
Age	0.02	(−0.07, 0.12)	0.07	(−0.21, 0.34)	0.00	(−0.04, 0.03)	0.15	
Sex	1.96	(−4.21, 8.12)	0.09	(−0.19, 0.36)	0.01	(−0.05, 0.03)	−0.01	
Previous experience ^a^	3.55 **	(1.06, 6.04)	0.40	(0.12, 0.68)	0.14	(−0.32, 0.03)	0.39 **	
								***R***^2^ = 0.165 *
**Step 2**								
(Intercept)	−0.99	(−25.07, 23.09)						
Warmth	0.29	(−0.42, 1.01)	0.13	(−0.19, 0.45)	0.01	(−0.06, 0.04)	0.10	
Emotional stability	−0.42	(−1.21, 0.38)	−0.17	(−0.50, 0.16)	0.02	(−0.08, 0.04)	−0.24	
Dominance	0.27	(−0.49, 1.03)	0.12	(−0.23, 0.47)	0.01	(−0.05, 0.03)	0.22	
Liveliness	−0.43	(−1.19, 0.32)	−0.19	(−0.51, 0.14)	0.02	(−0.09, 0.04)	−0.19	
Sensitivity	0.10	(−0.60, 0.80)	0.05	(−0.29, 0.39)	0.00	(−0.02, 0.02)	0.17	
Vigilance	−0.19	(−0.85, 0.47)	−0.08	(−0.39, 0.22)	0.01	(−0.04, 0.03)	−0.03	
Abstractedness	0.17	(−0.81, 1.14)	0.07	(−0.31, 0.44)	0.00	(−0.02, 0.02)	0.18	
Self-reliance	0.10	(−0.81, 1.01)	0.04	(−0.30, 0.37)	0.00	(−0.01, 0.01)	0.11	
Perfectionism	−0.47	(−1.25, 0.32)	−0.20	(−0.54, 0.14)	0.03	(−0.10, 0.04)	−0.15	
Tension	−0.46	(−1.27, 0.35)	−0.18	(−0.50, 0.14)	0.02	(−0.09, 0.04)	−0.03	
								*R*^2^ = 0.335

Note: A significant *b*-weight indicates the beta-weight and semi-partial correlation are also significant. *b* represents unstandardized regression weights. *beta* indicates the standardized regression weights. *sr*^2^ represents the semi-partial correlation squared. *r* represents the zero-order correlation. LL and UL indicate the lower and upper limits of a confidence interval, respectively. In the third step, only those significant interaction terms are reported. The intercept is omitted from the model. * indicates *p* < 0.05. ** indicates *p* < 0.01. ^a^ Previous experience was represented as a dummy variable, with 1 serving as meditation-naïve subject.

## Data Availability

The data that support the findings of this study are available from the corresponding author on reasonable request.

## Supplementary Materials

The following are available online at https://www.mdpi.com/article/10.3390/ijerph18136721/s1, Script: Reproducible R script, Table S1: Means, standard deviations and Pearson correlations, Table S2: No significant interaction results in Model 3 (interaction: intervention group) examining burnout reduction in main regression, Table S3: Intercept results and no significant interaction results in Step 3 (interaction: amount of home practice) examining burnout reduction for body-centered practices in secondary regression.
