# Supplementary material for: Interactions between Personality and Types of Mindfulness Practice in Reducing Burnout in Mental Health Professionals
Source: Int J Environ Res Public Health. 2021 Jun 22;18(13):6721. doi: 10.3390/ijerph18136721 (PMC8296896; doi:10.3390/ijerph18136721)
Supplement: Supplementary file 1 [file ijerph-18-06721-s001.zip › ijerph-1218028-supplementary.pdf]

SUPPLEMENTARY MATERIAL

Reproducible R script

Statistics were done using the emmeans (v 1.15-15; Lenth et al., 2020) and the apaTablet (v2.0.5; Stanley, 2018) packages.

```
#Main regression analyses#

---Step 1---

Step1_sindrome<-lm(Sindrome_dif~Edad+Sexo+Exp_previa, Base)

summary(Step1_sindrome)

apa.reg.table(Step1_sindrome, filename = "apa_reg_sindrome1.doc")#paquete
apa.tablet


---Step 2---

Step2_sindrome<-lm(Sindrome_dif~Edad+Sexo+Exp_previa

                  +Grupo+c_persA+c_persC+c_persE+c_persF+

                c_persI+c_persL+c_persM+c_persQ2+c_persQ3+c_persQ4,Base)

summary(Step2_sindrome)

apa.reg.table(Step2_sindrome, filename = "apa_reg_sindrome2.doc")


---Step 3---

Step3_sindrome<-lm(Sindrome_dif~+Edad+Sexo+Exp_previa+Grupo*c_persA+

               Grupo*c_persC+Grupo*c_persE+Grupo*c_persF+

                Grupo*c_persI+Grupo*c_persL+Grupo*c_persM+Grupo*c_persQ2

                     +Grupo*c_persQ3+Grupo*c_persQ4,Base)

summary(Step3_sindrome)

apa.reg.table(Step3_sindrome, filename = "apa_reg_sindrome3.doc")


#Simple slopes analyses#
```

```
mod<-lm(Sindrome_dif~Grupo*c_persQ3,Base)

emtrends(mod, ~ Grupo, var="c_persQ3")#paquete emmeans


#Secondary regression analyses#


Comparacion1<-filter(baseEM,Grupo!=2)#paquete dplyr

Comparacion2<-filter(baseEM,Grupo!=1)


---Step 1 burnout Group 1---

reg_3_1<-lm(Sind_diff~Edad+Sexo+Exp_previa,data = Comparacion1)

summary(reg_3_1)

apa.reg.table(reg_3_1,filename = "Sindrome_reg_3_1.doc")


---Step 2 burnout Group 1---

reg_3_2<-lm(Sind_diff~Edad+Sexo+Exp_previa+persA+persC+persE+persF+persI+
        persL+persM+persQ2+persQ3+persQ4,data = Comparacion1)

summary(reg_3_2)

apa.reg.table(reg_3_2,filename = "Sindrome_reg_3_2.doc")


---Step 3 burnout and informal practice---

reg_3_3a<-lm(Sind_diff~persC:Prac_3+persL:Prac_3+persQ2:Prac_3,data =
Comparacion1)

summary(reg_3_3a)

apa.reg.table(reg_3_3a,filename = "Sindrome_reg_3_3a.doc")


---Step 3 burnout and formal practice---
```

```
reg_3_3b<-lm(Sind_diff~persC:Prac_4+persL:Prac_4+persQ2:Prac_4,data =
Comparacion1)

summary(reg_3_3b)

apa.reg.table(reg_3_3b,filename = "Sindrome_reg_3_3b.doc")


---Step 1 burnout Group 2---

reg_4_1<-lm(Sind_diff~Edad+Sexo+Exp_previa,data = Comparacion2)

summary(reg_4_1)

apa.reg.table(reg_4_1,filename = "Sindrome_reg_4_1.doc")


---Step 2 burnout Group 2---

reg_4_2<-lm(Sind_diff~Edad+Sexo+Exp_previa+persA+persC+persE+persF+persI+
        persL+persM+persQ2+persQ3+persQ4,data = Comparacion2)

summary(reg_4_2)

apa.reg.table(reg_4_2,filename = "Sindrome_reg_4_2.doc")
```

Suplementary references

Lenth, R., Singmann, H., Love, J., Buerkner, P., & Herve, M. (2020). *Package ' emmeans .'* R Package Version 1.15-15. https://doi.org/10.1080/00031305.1980.10483031>.License

Stanley, D. (2018). *apaTables: Create American Psychological Association (APA) Style Tables. R package version 2.0.5*. https://cran.r-project.org/package=apaTables

Suplementary tables

Table S1. Means, standard deviations and Pearson correlations.

	**2.**	**3.**	**4.**	**5.**	**6.**	**7.**	**8.**	**9.**	**10.**	**11.**	**12.**	**13.**	**14.**
1.Burnout	−0.025	−0.220**	0.245*	−0.106	−0.024	−0.249*	0.119	0.178	−0.163	−0.026	−0.088	−0.038	0.025
2.16PF-Warmth	-	−0.076	−0.122	−0.125	−0.123	0.182	0.072	0.225*	−0.216*	−.073	−0.053	−0.096	-0.057
3. 16PF-Emotional stability		-	0.133	0.009	0.075	0.163	0.049	−0.121	0.002	0.294**	−0.144	−0.098	−0.068
4. 16PF-Dominance			-	−0.107	−0.015	0.048	−0.040	−0.140	0.217*	−0.147	−0.083	0.135	0.053
5. 16PF-Liveliness				**-**	0.106	0.081	0.038	0.114	−0.079	0.078	0.024	−0.028	0.027
6. 16PF-Sensitivity					-	0.054	0.082	0.007	0.032	−0.269**	−0.135	−0.261*	0.159
7. 16PF-Vigilance						-	−0.098	−0.025	0.081	−0.049	−0.057	−0.173	0.052
8. 16PF-Abstracted-ness							-	0.064	0.108	−0.104	0.056	0.160	0.082
9. 16PF-Self-reliance								-	0.021	0.010	−0.155	−0.081	<-0.001
10. 16PF-Perfection-ism									-	0.032	−0.005	0.025	−0.076
11. 16PF-Tension										-	−0.096	−0.138	0.062
12. Formal practice											-	−0.573**	−0.123
13. Informal practice												-	<-0.001
14. Age													-
Mean *(SD)*	5.87 (2.00)	5.65 (1.7)	7.38 (1.97)	6.17 (1.80)	5.89 (2.00)	6.16 (1.99)	6.28 (1.70)	5.88 (1.57)	5.67 (1.76)	4.31 (1.97)	300.8 (192.6)	238.6 (168.1)	

NOTE: 16 PF = Cattell´s 16 Personality Factors Test. ** $p < 0.01$. * $p < 0.05$.

Table S2. No significant interaction results in Model 3 (interaction: intervention group) examining burnout reduction in main regression.

	b	*b* 95% CI [LL, UL]	*sr*²	*sr*² 95% CI [LL, UL]
**Model 3**				
Group x Warmth	−0.15	[−1.17, 0.87]	0.00	[−0.01, .01]
Group x Emotional stability	0.64	[−0.40, 1.69]	0.01	[−0.02, .05]
Group x Dominance	0.57	[−0.37, 1.52]	0.01	[−0.02, .04]
Group x Liveliness	−0.22	[−1.10, 0.66]	0.00	[−0.01, .02]
Group x Abstractedness	0.67	[−0.40, 1.74]	0.01	[−0.02, .05]
Group x Self-reliance	−0.78	[−2.03, 0.47]	0.01	[−0.02, .05]
Group x Perfectionism	0.22	[−0.94, 1.38]	0.00	[−0.01, .01]

NOTES: *b* represents unstandardized regression weights; *LL* and *UL* indicate the lower and upper limits of a confidence interval, respectively.

Table S3. Intercept results and no significant interaction results in Step 3 (interaction: amount of home practice) examining burnout reduction for body-centered practices in secondary regression

	b	*b* 95% CI [LL, UL]	*sr*²	*sr*² 95% CI [LL, UL]
**Step 3**				
(Intercept)	1.69	[−0.34, 3.72]		
Emotional stability x informal practice	−0.00	[−0.00, 0.00]	0.03	[−0.13, −0.07]
(Intercept)	2.19	[−0.07, 4.44]		
Emotional stability x formal practice	−0.00	[−0.00, 0.00]	0.03	[−0.13, 0.07]
Vigilance x formal practice	−0.00	[−0.00, 0.00]	0.07	[−0.23, 0.08]
Self-reliance x formal practice	0.00	[−0.00, 0.00]	0.10	[−0.28, 0.08]

NOTES: *b* represents unstandardized regression weights; *LL* and *UL* indicate the lower and upper limits of a confidence interval, respectively
